# Health Information and Monitoring of Sexually Transmitted Infections (SIM study): a single-center, parallel, three-arm randomized controlled trial protocol for enhancing adherence to syphilis treatment and follow-up

**DOI:** 10.1186/s13063-022-06383-w

**Published:** 2022-05-26

**Authors:** Eliana M. Wendland, Vanessa M. de Oliveira, Luana Giongo Pedrotti, Flavia M. A. Souza, Gerson F. M. Pereira, Antonio Gerbase

**Affiliations:** 1 Hospital Moinhos de Vento, Porto Alegre, Brazil; 2 Graduate Program in Health Sciences and Pediatrics, Federal University of Health Sciences of Porto Alegre, Porto Alegre, Brazil; 3 Department of Chronic Conditions Diseases and Sexually Transmitted Infections, Ministry of Health, Brasília, Distrito Federal Brazil

**Keywords:** Syphilis, Clinical trial, Telemonitoring, Treatment adherence, Protocol, Randomized controlled trial

## Abstract

**Background:**

Syphilis has recently resurfaced as a significant public health problem. Since the 2000s, isolated syphilis outbreaks have increasingly occurred in North America, Europe, and Australia; in Brazil, there have been progressive increases in both congenital and acquired syphilis. There are several possible explanations, such as misdiagnosis of acquired syphilis, which could increase the number of untreated transmitters in the population; failure to initiate or complete treatment; and nontreatment of sexual partners (leading to reinfection). Mobile technologies have been successfully used to promote behavior changes and can positively impact treatment and follow-up adherence in patients with infectious diseases. The purpose of this clinical trial is to evaluate treatment and monitoring methods in patients with syphilis, including follow-up by telephone, via a game in a smartphone app, and at public health centers.

**Methods:**

The SIM study is a single-center, randomized controlled trial with a 12-month follow-up period. The aim is to identify the most effective method of follow-up regarding patient compliance with treatment. The tests will be performed in a mobile unit in easily accessible locations. The goal is to perform 10,000 rapid tests for syphilis. Patients with a confirmed diagnosis according to VDRL tests will be randomized to one of three arms: telephone, smartphone game, or conventional in-person follow-up. All analyses will follow the intention-to-treat principle.

**Conclusion:**

If we find differences in effectiveness, a major change in the conventional approach for this patient population may be needed, potentially affecting current Brazilian health policy strategies.

**Trial registration:**

NTC04753125. Version 1 of protocol 1/09/2020.

**Supplementary Information:**

The online version contains supplementary material available at 10.1186/s13063-022-06383-w.

## Introduction

Syphilis infection has recently re-emerged as a significant public health problem, with an increasing incidence in many countries [1]. Syphilis is a bacterial infection caused by *Treponema pallidum*; transmission occurs through sexual contact (acquired syphilis) or during pregnancy (vertical transmission) [2]. The primary lesion (primary syphilis) frequently goes unnoticed, and many individuals do not notice any symptoms during the early stages of infection. Congenital syphilis, on the other hand, occurs when a mother infected with syphilis transmits the infection to her child during pregnancy; in these cases, the disease has a poor prognosis, and the risks of spontaneous abortion, fetal death, and premature infant death are increased [3].

Beginning in the 2000s, a growing number of isolated syphilis outbreaks were reported in North America, Europe, and Australia, primarily within sexual networks of men who have sex with men (MSM); syphilis infection is often associated with HIV coinfection and cotransmission [4]. While the number of cases in specific groups has increased in high-income countries, in low-middle-income countries, syphilis remains an endemic disease, affecting the general population [5].

In Brazil, there has been a progressive increase in both congenital and acquired syphilis in recent years. Data from compulsory reports indicated that acquired syphilis rates increased from 2.0 cases/100,000 inhabitants in 2010 to 58.1 cases/100,000 inhabitants in 2017. Congenital syphilis also increased substantially (3.6-fold) from 2.4 to 8.6 cases per 1000 live births. Although these increases occurred in all Brazilian regions, some geographical regions had rates above the Brazilian average, including the Southern Region, in which the city of Porto Alegre had an incidence rate of congenital syphilis 3.8 times higher than the rate in the Brazilian general population [6, 7].

Several causes could be implicated in the increasing number of cases, such as misdiagnosis of acquired syphilis, which could lead to a larger number of untreated transmitters in the population; failure to initiate or complete treatment; nontreatment of sexual partners (leading to reinfection); or lack of adequate prenatal care, as syphilis is most often diagnosed during pregnancy screening or during screening for other sexually transmitted infections [7].

There are 8.3 billion mobile phone subscriptions worldwide; this number is greater than the global population, and the numbers of subscribers in low- and middle-income countries are increasing rapidly, thereby increasing opportunities to easily connect with a large number of patients to provide individual-level support [8]. Telemedicine by phone has increased widely in the country and in different areas. Although telemedicine has been shown to promote follow-up adherence to treatment protocols for chronic conditions [9–11], there is no evidence of its impact on STIs. Additionally, there is pressure in the country for its implementation. Regularly followed patients were chosen as the control group.

Herein, we describe the study protocol for a parallel randomized controlled trial to evaluate different home telemonitoring technologies to enhance adherence to treatment and follow-up in syphilis patients.

Mobile technologies and strategies to track test results and appointments have been successfully used as tools to promote behavior changes and can positively impact adherence to treatment and follow-up in patients with chronic [9, 12–14] and infectious diseases [10, 11, 15, 16]. The main hypothesis is that participants who are allocated to the game will have greater adherence to syphilis treatment and monitoring than contacts with telephone contact, and both intervention arms will have better adherence to treatment and follow-up than those randomized to conventional attention. The app group will be compared to the telephone calls and conventional attention groups, and the telephone call arm will be compared to the conventional attention.

## Materials and methods

### Study design

The SIM study is a single-center, superior, randomized controlled trial with a 1:1:1 allocation ratio that includes three parallel arms: (1) follow-up with telephone calls, (2) follow-up using gamification in a mobile app, and (3) follow-up with usual care provided by the public health system. The conventional strategy in Brazil involves through mandatory notification by the physician and guidance to the patient, but it does not consist of an active search through home visits or telephone contacts. Randomization will occur at the individual level.

### Participants and recruitment

All adults aged 18 years and older will be invited to answer a questionnaire to collect socioeconomic and sexual behavior data and undergo rapid tests for syphilis, HIV, and hepatitis B and C in a mobile unit located in an area accessible to a large population. The unit will have different attractive features, such as free internet, charging areas for smartphones, and shaded and rest areas with screens, to increase the participation rate. All participants will receive an ecobag plus preservatives and lubricants. Participants with a positive rapid test will be invited to undergo confirmatory testing. This protocol was written following the Standard Protocol Items: Recommendations for Interventional Trials (SPIRIT) statement to define the clinical trial protocol.

### Eligibility criteria

The study will be carried out in Porto Alegre, Southern Brazil. Participants who do not return after three contact attempts will be excluded from the study, as will pregnant women, participants who are not able to provide contact information, participants who are illiterate and those who underwent syphilis treatment within the previous 3 months.

### Sample size

Considering a confidence level of 95%, an incidence rate of 93.7 per 100,000 inhabitants in the municipality of Porto Alegre [9], and an acceptable difference of 60.0 per 100,000 inhabitants, it is estimated that testing 10,000 individuals will be necessary to obtain an accurate prevalence of syphilis in the municipality. This sample will serve as the basis for the inclusion of patients in the clinical trial.

For the randomized clinical trial, another sample size calculation will be considered. Using a previous article in which 16.5% of the participants who were diagnosed with syphilis received appropriate treatment and 55.9% received inappropriate treatment as a reference, it is expected that at least half of the latter group will eventually receive proper treatment, for a total of 44.45% receiving appropriate treatment [17]. Thus, considering a sample size calculation to detect differences in the proportions of adequate treatments between groups, using a 95% confidence level, 80% power, and a maximum loss rate of 20%, 51 participants in each study group will be needed.

The primary goal of the study is to compare treatment adherence and follow-up among the three parallel study arms. The groups will be compared as a whole, and if relevant, pairwise analysis will be conducted. Such comparisons will be performed using the chi-square test and adjusted residuals when necessary. We will investigate the association between treatment

adherence and sociodemographic, sexual health, and sexual behavior variables as secondary outcomes. The association will be estimated by calculating crude and adjusted odds ratios (ORs) using logistic regression, also addressing confounding variables. Intention-to-treat analysis will be used to assess the clinical effectiveness of the intervention.

We anticipate a minimal number of missing values given that the study variables will be collected by trained researchers. However, data quality analysis will be applied to identify inconsistencies and missing data, and meetings with the researchers will be conducted to discuss missing data and prevent further inconsistencies.

A statistical significance level of 0.05 will be adopted for all statistical analyses. R software, version 4.0.3, will be used for the analysis.

### Study procedures

A paper-based questionnaire to collect sociodemographic, behavioral, and health information will be administered to all participants to check eligibility, and a rapid test for syphilis will be performed. All participants with a positive test result will undergo confirmatory testing. Upon the receipt of confirmatory test results, the participants will be invited to take part in the clinical trial, and a web-based questionnaire will be used to collect information such as age, sex, social class, education level, alcohol consumption, tobacco and drug use, risk for syphilis, history of hepatitis and other sexually transmitted infections (STIs), number of sexual partners, type of sex and condom use. The main objective of the trial is to test strategies to improve adherence to follow-up. Overall, to increase awareness regarding syphilis, all participants will receive informative folders on syphilis, HIV, and hepatitis prior to randomization. Adherence will be monitored by the number of accesses to the application and by successful telephone calls.

#### Randomization and blinding

Patients will be randomized at a ratio of 1:1:1 among the three intervention arms: follow-up by telephone, follow-up using a smartphone game, or follow-up with usual care in the public health system. Random allocation will be performed in blocks with variable sizes, stratified by participants with and without smartphone access. Randomization will be performed using R software [18]. Individuals who have access to a smartphone will be randomly assigned to any of the 3 groups, while individuals without access to a smartphone will be randomly assigned to one of only two groups: the telephone follow-up group or the control group.

An independent statistician will be responsible for computerized randomization (R software [18]) but will not be involved in the determination of participant eligibility, assessment or implementation of the interventions. Blinding will be applied to minimize bias. Allocation concealment as the person responsible for the randomization will be applied to the randomized group only after the participants have been recruited, which will occur after baseline measurements are completed. Researchers responsible for the follow-up by telephone intervention will not have access to performance and evaluation data. Only the biostatistician will have access to the data stored in the data entry system, minimizing investigator influence.

#### Intervention

**Fig. 1:**
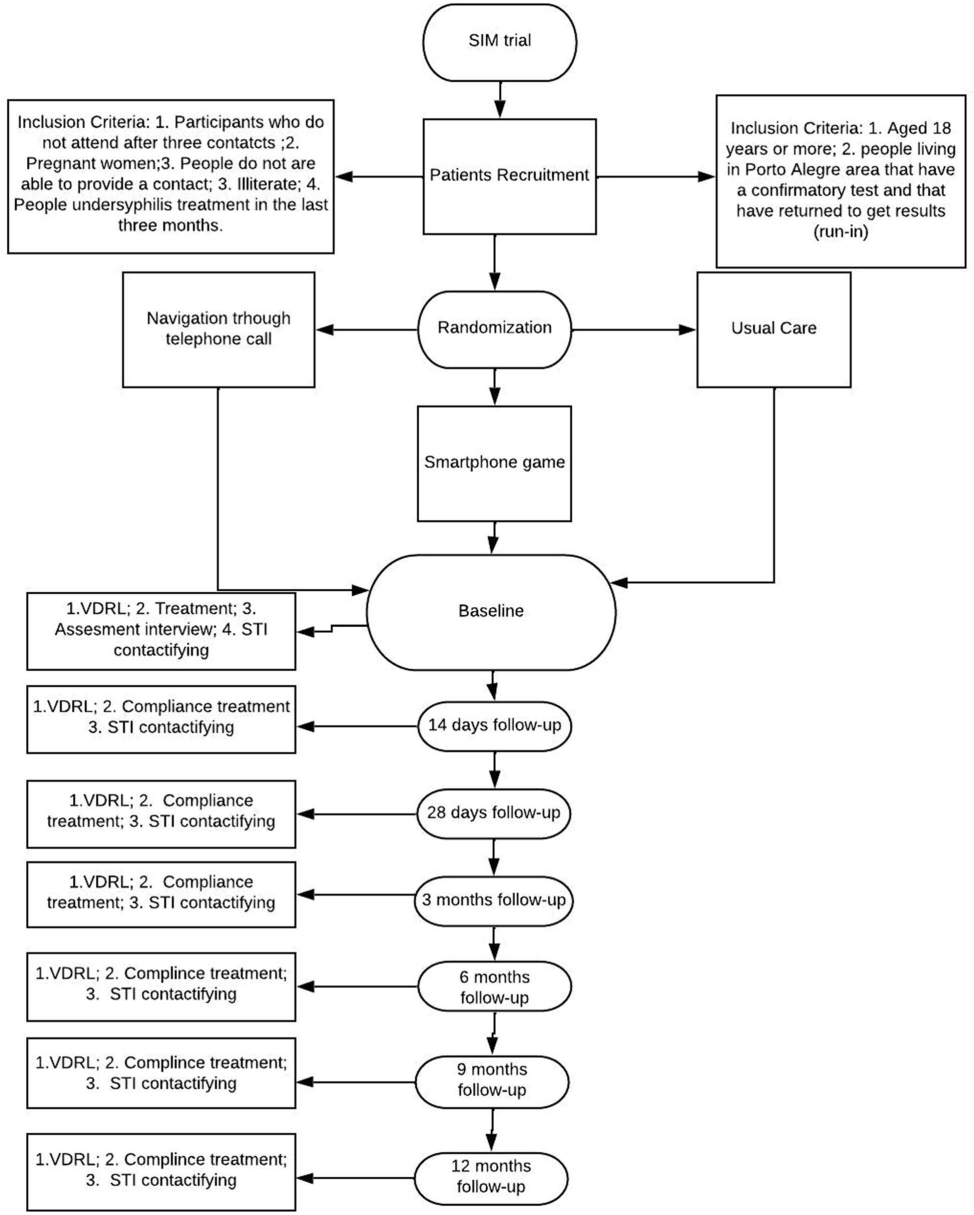
Profile of study

The intervention strategies aim to increase adherence to treatment and monitoring and promote communication with sexual partners (Fig. 1). All intervention and conventional strategies followed the guidelines of the Clinical Protocol and Therapeutic Guidelines for Comprehensive Attention to People with Sexually Transmitted Infections of the Ministry of Health of Brazil [4].

#### Strategy 1: Follow-up by telephone

Initial contact will be made 2 days before treatment, on the day of treatment and for 14 days until the next treatment dose. If the patient fails to apply treatment within 14 days of the last treatment dose, the intervention will be considered a failure, and the patient will have to restart treatment. For monitoring, contact will be made 7 days before the exam and on the day of the exam to encourage the patient to get tested, and the patient will receive 1 call per week for 3 weeks until the patient takes the VDRL test.

#### Strategy 2: Follow-up using a game in a smartphone app

A thematic game will be developed to encourage treatment and monitoring in patients with syphilis. The game will be easy, similar to other commercial games such as Candy Crush (King), Bubble Shoote (Sukavaty), Block Puzzle (Honkong Sanyou), and Block Puzzle Jewel-puzzle game (Elimination Master) and can be played by different ages and different education levels. The first phase of the game will be open to all study participants regardless of their rapid test results and will have information about prevention strategies and sexually transmitted infections. In the second phase, the participants will be prompted by notifications via smartphone 2 days before the day of treatment, on the day of treatment, and every day for 14 days after the date they should have received the injection. The participant is awarded coins to buy clothing and accessories for his or her avatar if he or she reads the information about STIs and provides information about his or her partners. The participant passes the phase of the game when he or she has taken the medication doses and completed the VDRL test. In different game phases, the avatar takes the bus and travels to different capitals of Brazil, where syphilis infection rates are also high.

#### Strategy 3: Conventional follow-up

The conventional strategy involves compulsory notification by a healthcare professional, and the patient is directed to seek treatment and follow-up at the primary health care unit closest to his or her home. The outcomes will be accessed by reviewing data from the public health records.

### Outcome assessment

Compliance with treatment for those in the mobile app arm will be assessed according to the uploaded game data provided by the participant and use of the app during the study period. Adherence to treatment for those receiving telephone follow-up will be assessed through routine phone calls and questionnaire questions about the date of examination and treatment. Adherence in the usual care arm will be assessed by information collected from the participant's medical record at their referral health unit. The primary outcome will be the percentage of the participants who finish all three doses of penicillin in the 3 weeks. The secondary outcome will be the percentage of participants who completed VDRL tests at 3, 6, and 12 months. We also evaluated the number of sexual contacts informed by each participant. The outcomes will be evaluated by self-declaration and by contacting the healthcare unit where the participants made treatment and follow-up.

### Specimen processing

Samples will be treated as biohazardous material, and specimen handling will be carried out in a biosafety cabinet. Once arriving in the laboratory, serum samples will be immediately fractionated. Two aliquots will be frozen at − 80°C as backup samples. All samples with positivity on the treponemal test (rapid test) will be subjected to a confirmatory nontreponemal test. The aliquot will be directly used for the VDRL and rapid plasma regain (RPR) tests. If the VDRL test is negative, an additional treponemal test will be used to confirm (TPHA) syphilis positivity. An online platform for data entry will be used by the study team to add participant data, rapid test results, biological sample information, and exam reports. Data will be monitored daily by the project statistician.

### Statistical analyses

Descriptive analysis will be performed to characterize the study population. The categorical variables will be summarized by absolute frequencies and percentages, while continuous variables will be described as the means and standard deviations or medians and interquartile ranges.

To compare proportions, the chi-square test and Fisher's exact test will be used. Student’s *t* test or the nonparametric Mann–Whitney test will be used to compare continuous variables.

The primary goal of the study is to compare treatment adherence and follow-up among the three parallel study arms. We will investigate the association between treatment adherence and sociodemographic, sexual health, and sexual behavior variables as secondary outcomes. The association will be estimated by calculating crude and adjusted odds ratios (ORs) using logistic regression. Intention-to-treat analysis will be used to assess the clinical effectiveness of the intervention.

A statistical significance level of 0.05 will be adopted for all statistical analyses. R software, version 4.0.3, will be used for the analysis [18].

### Monitoring and quality control

During the project development stage, operational manuals were developed along with the questionnaires to serve as general guidelines for conducting interviews, standardizing information, and solving general doubts. All researchers involved in the data collection will be trained using simulated interviews and biological sample collections.

A pilot study will be performed for one month to identify and correct problems with the interview, logistical and study procedures.

The participants will be informed about the study results through reports that will be provided at the mobile unit of the study.

Quality control will be performed through system reports, data entry control, and data enrollment auditing. During the project development stage, we established written policies for sample management and quality control in the laboratory handbook. All laboratory professionals were trained and certified by the coordinator team according to the study protocol. Those performing the VDRL tests are certified by the National Program of Quality Control (PNCQ).

A web-based platform will be utilized for biological sample tracking and test result recording on a daily basis. Samples will be carefully transported to maintain the integrity of the sample, with attention to temperature, preservation methods, special transport containers, and time limitations. Once the samples arrive in the laboratory, their label information, quantity, and condition for the requested tests will be verified. Frozen specimen aliquots will be tracked using the Freezer Web system. Test results will be photo-documented, and the images will be uploaded to a digital repository.

We will perform an interim analysis only at 6 months after the start of the randomization to assess adaptations such as sample size recalculations and changes in eligibility criteria. Additional analyses for study interruption will not be necessary due to the low risk of the intervention being applied. Additionally, we expect no adverse effects of this intervention.

#### Ethics and dissemination

The study was approved by the Research Ethics Board (REB) of the Hospital Moinhos de Vento and Health Secretariat of the Municipality of Porto Alegre. Written consent will be obtained from all participants after they have been informed about the study procedures. All participants will provide informed consent addressing the questionnaire and the storage and use of biological samples. Data entry will be protected by personal logins and will be available to only the main statistician after the interview is finalized. Authorship eligibility followed the CRediT guidelines.

### Steering committee

The study will not have a monitoring committee due to the nature of the intervention, which is of minimal risk to participants.

### Dissemination

The results of the study will be disseminated to the different stakeholders by providing reports to the Ministry of Health and state and federal government agencies, publishing articles in scientific journals, and presenting the results at scientific conferences and to the Municipal Health Committee, of which users are part of the Single Health System.

## Discussion

Syphilis has been diagnosed in laboratories for over 100 years. Testing is becoming increasingly cheaper, faster, less invasive, and easier. Treatment for this disease has been known for over 60 years, and penicillin is a cheap drug with no major adverse events. However, syphilis has been re-emerging in Brazil and in many countries around the world. The World Health Organization (WHO) estimates that 11 million new cases of syphilis occur each year in Latin America, with 70% occurring in Brazil.

Open syphilis lesions facilitate HIV transmission. Thus, preventing and treating syphilis could also reduce HIV transmission. Furthermore, coinfection with HIV may change the natural course of syphilis.

The increase in the number of syphilis cases has resulted in new challenges for health management, and new data to guide effective interventions to control the disease are needed. Some questions need to be answered so that better prevention and control strategies can be developed and perhaps generate new health policies. Our study intends to answer the following questions: What is the better e-Health strategy for enhancing treatment and monitoring in patients with syphilis? Will any of the interventions increase the communication with and treatment of sexual partners?

## Supplementary Information

**Additional file 1.**

## Data Availability

The datasets used and/or analyzed during the current study are available from the corresponding author on reasonable request. Quality control will be performed through system reports, data entry control and data enrollment auditing. During the project development stage, we established written policies for sample management and quality control in the laboratory handbook. All laboratory professionals were trained and certified by the coordinator team according to the study protocol. Those performing the VDRL tests are certified by the National Program of Quality Control (PNCQ).
