# Supplementary material for: Health Information and Monitoring of Sexually Transmitted Infections (SIM study): a single-center, parallel, three-arm randomized controlled trial protocol for enhancing adherence to syphilis treatment and follow-up
Source: Trials. 2022 May 26;23:445. doi: 10.1186/s13063-022-06383-w (PMC9137047; doi:10.1186/s13063-022-06383-w)
Supplement: Supplementary file 1 — Additional file 1. [file 13063_2022_6383_MOESM1_ESM.docx]

| **Data category** **Information** |
| --- |
| **Primary registry and trial identifying number ClinicalTrials.gov**: NCT 04753125 |
| **Date of registration in primary registry**: 1/09/2020 |
| **Secondary identifying numbers**: |
| **Source(s) of monetary or material support**: Institutional Development of the Public Health System (PROADI-SUS) and supported by the Brazilian Ministry of Health. |
| **Primary sponsor:** Institutional Development of the Public Health System (PROADI-SUS) |
| **Secondary sponsor(s):** Brazilian Ministry of Health |
| **Contact for public queries:** [elianawend@gmail.com](mailto:elianawend@gmail.com) |
| **Contact for scientific queries:** [elianawend@gmail.com](mailto:elianawend@gmail.com) |
| **Public title:** Health Information and Monitoring of Sexually Transmitted Infections (SIM Study): a single-center, parallel, three-arm randomized controlled trial protocol for enhancing adherence to syphilis treatment and follow-up |
| **Scientific title:** Health Information and Monitoring of Sexually Transmitted Infections (SIM Study): a single-center, parallel, three-arm randomized controlled trial protocol for enhancing adherence to syphilis treatment and follow-up |
| **Health condition(s) or problem(s) studied:** Syphilis, adherence of treatment |
| **Intervention(s):** |
| Active comparator: game |
| Placebo comparator: call telephone and conventional strategy |
| **Key inclusion and exclusion criteria:** |
| Inclusion criteria: Ages eligible for study: ≥18 years; Sexes eligible for study: both, VDRL test positive. |
| Exclusion criteria: Participants who do not return after three contact attempts will be excluded from the study, as well as pregnant women, participants who are not able to provide contact information, participants who are illiterate and those who underwent syphilis treatment within the previous three months. |
| **Study type:** |
| Interventional Allocation: randomized; |
| Intervention model: parallel assignment; |
| Masking: simple blind |
| Primary purpose: prevention Phase III |
| **Date of first enrolment:** January 2022 |
| **Target sample size:** 51 |
| **Recruitment status:** Recruiting |
| **Primary outcome(s)**: Game better adherence in the treatment |
| **Key secondary outcomes:** Prevalence of syphilis |
